# Evaluation of the Efficacy of Buccal Midazolam in Comparison With Intranasal Midazolam Sedation in Uncooperative Children During Dental Treatment

**DOI:** 10.1155/ijod/4269519

**Published:** 2025-03-17

**Authors:** Doaa Arnaout, Mohamed Altinawi, Imad Katbeh, Nikolay Tuturov, Ahmad Saleh

**Affiliations:** ^1^Faculty of Dental Medicine, Department of Pediatric Dentistry, Damascus University, Damascus, Damascus Governorate, Syria; ^2^Department of Pediatric Dentistry and Orthodontics, RUDN University, 6 Miklukho-Maklaya Street, Moscow 117198, Russia

**Keywords:** buccal administration, child behavior, intranasal administration, midazolam/administration and dosage, sedation, uncooperative children

## Abstract

**Aim:** Behavioral management techniques are not always sufficient, and then it is necessary to use pharmacological management methods. The aim of this study is to compare the effectiveness of buccal midazolam sedation with intranasal midazolam in non-cooperative children during dental treatment.

**Materials and Methods:** A randomized single blinded comparative clinical study consisted of 40 children aged 3–6 years who were divided randomly into two groups: Group A intranasal midazolam and Group B buccal midazolam. The onset time of action and recovery time from sedation were compared between the two groups, and the efficacy of sedation was evaluated by Houpt behavior scale. The independent student's *T* test, Mann–Whitney *U* test, the Wilcoxon test and the Chi-square test were used.

**Results:** There were no statistically significant differences in the onset time of action (*p*=0.458) and recovery time from sedation (*p*=0.148). There were no statically significant differences between the two groups in sleeping, crying, and movement categories (*p*=0.747), (*p*=0.183), (*p*=0.732), respectively, or in the overall Houpt scale (*p*=0.393), there were statistically significant differences in the sleep variable between the two studied phases in the intranasal group (*p*=0.014) and in the movement variable in the buccal group (*p*=0.039).

**Conclusion:** Both buccal midazolam and intranasal sedation were effective in the management of uncooperative children during dental treatment at 85% and 80%, respectively.

**Trial Registration:** Australian New Zealand Clinical Trials Registry: ACTRN12624000945527

## 1. Introduction

Dental anxiety has multiple causes, making it hard to be managed in a single way for all patients [1]. If behavior management techniques do not work, pharmacological management methods, such as sedation and general anesthesia, should be employed [2]. Moderate sedation (previously known “conscious sedation”) is defined as: a drug-induced depression of consciousness during which patients respond purposefully to verbal commands or after light tactile stimulation. Maintaining a patent airway is achievable without any interventions, spontaneous ventilation is sufficient. Cardiovascular function is typically maintained [3]. Midazolam is the most widely used benzodiazepine for promoting a safe and effective sedation experience without the risk of cardiopulmonary complications [4]. Transmucosal sedation (nasal, sublingual, and buccal) has recently received a lot of attention in pediatric dentistry [5–8]. Intranasal midazolam can effectively improve general behavior in children and considerably reduce unwanted movements, in addition to having a rapid onset of action [9–12]. Intranasal midazolam can cause discomfort because it irritates the nasal mucosa, leading to coughing and sneezing, which can expel some of the drug [13]. The oral cavity is a proper site for systemic medication delivery as the oral mucosa is potent, has a high blood supply, and offers short recovery periods after trauma or harm [6]. The usage of buccal midazolam sedation is uncommon in pediatric dentistry, yet it is more tolerable and acceptable for children than intranasal sedation [7, 14].

The aim of this study is to compare the effectiveness of buccal midazolam sedation with intranasal midazolam in uncooperative children during dental treatment.

## 2. Materials and Methods

The study was designed as a comparative, randomized single blinded clinical trial, to assess the efficacy of transmucosal midazolam sedation in uncooperative children during dental treatment. The study protocol was accepted by the Scientific Research and Postgraduate Board of Damascus University, Ethics Committee of Damascus University, Damascus, Syria (IRB number: UDDS-3232-07092020/SRC-631). The trial has been retrospectively registered. Registration date is 05/08/2024. The sample size included 40 patients, their ages ranged between 3 and 6 years, who were randomly divided into two groups, group A: 0.3 mg/kg intranasal midazolam sedation, group B: 0.3 mg/kg buccal midazolam sedation. The randomization was done by the randomization tables on excel.

### 2.1. Inclusion Criteria

The patient is classified as ASA (I and II), patient's behavior is within the second degree (negative) of the Frankle scale, and the patient needs a dental treatment which includes pulpotomy for a lower molar.

### 2.2. Exclusion Criteria

A patient who refused to participate in the study, the known hypersensitivity to midazolam, upper respiratory tract infection or tonsillar hypertrophy, children with aggressive behavior or high resistance to treatment.

After ensuring that patient meets with inclusion criteria, the written informed consent of parents or guardians was obtained. Fasting instructions before treatment session were given in the following form: clear liquids 2 h, human milk 4 h, infant formula, nonhuman milk, light meal 6 h.

On the day of treatment, the child's weight and vital signs were recorded. The children in the two groups received undiluted midazolam (Dormita-5, Al-saad company, Damascus, Syria) (5 mg/mL) in a dose of 0.3 mg/kg. In group A, intranasal midazolam was sprayed into both nostrils using a mucosal atomizing device (MAD) (MAD 300, Teleflex medical Inc., USA). In group B, midazolam was sprayed in the buccal sulcus using the MAD after applying lips retractor and drying the mucosal membrane. High-speed suction was applied on the floor of the mouth for 2 min which allowed for achieving maximum absorption through mucosa. The onset time of action was recorded in minutes, the level of sedation was considered appropriate for treatment when the patient seemed relaxed or when he showed delay in speech and in response to commands. After application of topical anesthesia (benzocaine 20%, DHARMA RESEARCH, USA), local anesthesia was administrated using 27-gauge short needles and syringes loaded with carpules, each one contained 1.8 mL of Lidocaine 2% with epinephrine concentration of 1 : 80,000 (New Stetic S.A., Colombia). Complete isolation was performed using a rubber dam and saliva ejector. Removal of caries and deroofing of the pulp chamber were performed using a no. 330 high-speed carbide bur with copious water spray. A slow-speed round carbide bur (no. 6) was used for coronal pulp amputation. Then, the pulp chamber was washed with normal saline and bleeding was controlled by placing a cotton pellet moistened with water in the pulp chamber for 5 min, a sterile cotton pellet moistened with 1 : 5 concentration formocresol (Buckley's Formocresol, Sultan Healthcare, Englewood, NJ, USA) then blotted to remove excess was placed for 5 min on the pulp stumps and then the pulps were covered with zinc oxide–eugenol (IRM; Dentsply, Milford, DE) dressing, the tooth finally was restored using a stainless steel crown (SSC) (3M/ESPE, St. Paul, MN, USA) [15].

The behavioral response was assessed using Houpt behavioral scale of sleep, crying, and movement (Table 1). The treatment was recorded and the variables were evaluated in two phases, the first phase is at the moment of local anesthesia, the second phase is after 15 min from anesthesia. The blindness was applied to the examiners who assessed the Houpt scale scores, the treatment was recorded and then the videos were rewatched by three examiners specialists in pediatric dentistry (having a master's degree in pediatric dentistry) after training them on the used scale, the scale score was accepted when at least two examiners agreed, with the assurance that the examiners did not know the used method of sedation. The general behavior was assessed at the end of treatment using the overall Houpt rating scale, the success rate of treatment was evaluated in which one and two grades were considered failure of the treatment and the other four grades were considered success of the treatment. The recovery time from sedation was recorded in minutes from the point of end of treatment, till the patient became fully conscious, fully calm, and responded to the verbal commands effectively [16].

Data were collected and analyzed using SPSS v.13 with a significance level of 0.05. The independent student's *T* test was used to study the difference in the mean of onset time of action, and recovery time from sedation between the two groups.

The Mann–Whitney *U* test was used to study the difference in the grades of (sleep, crying, and movement) between the two groups in the two studied phases (at local anesthesia, after 15 min), and to study the difference in the HOUPT general behavior scale between the two groups. The Wilcoxon test was used to study the difference in the grades of (sleep, crying, and movement) between the two studied phases according to the method of sedation. The Chi-square test was used to study the difference in the result of sedation success between the two groups.

The null hypothesis:

• There are no statistically significant differences between the median of the recorded parameters variables between the buccal midazolam and intranasal midazolam sedation (onset time of action and recovery time from sedation).
• There are no statistical differences between the repetitions of the grades of the nominal variables between the buccal and intranasal midazolam groups (the grades of Houpt scale “sleeping, crying, and movement,” the success rate of sedation).
• There are no statistical differences between the repetitions of the grades of the nominal variables between the two studied stages in each buccal and intranasal midazolam sedation group.

## 3. Results

The research sample involved 40 children aged between 3 and 6 years, with an average of (4.8 ± 0.9 years).

There was no significant difference in the mean of the onset of action between the groups (*p* value = 0.458). There was no significant difference in the mean of the recovery time from sedation (*p* value = 0.148).

There was no statistically significant difference in the frequency of sleeping grades between the two groups. However, statistically significant differences were found in the intranasal midazolam group when comparing the frequency of sleep between the two studied stages (anesthesia, after 15 min), 65% of the children were drowsy during anesthesia and the percentage increased to 95% in the second stage, and the rest of the children were at the stage of “fully awake” in both stages without any of them reaching the stage of deep sleep. As for the buccal midazolam sedation, only one child reached the stage of “sleepy and difficult to wake” in the second stage of treatment, and the rest of the children were distributed between the stages of fully awake and drowsy with the tendency to get asleep to a greater degree 60% and 65% in the first and second stages, respectively, without statistically significant differences (Table 2).

There was no statistically significant difference in the repetitions of the degree of crying between the two groups, or between the two studied stages (anesthesia, after 15 min), regardless of the used sedation method. In total, 40% of the children in both groups were crying continuously during anesthesia, and 15% of the children in the intranasal group were crying hysterically. The children cried hysterically in the second stage “after 15 min” in intranasal and buccal midazolam in the percent of 30% and 10%, respectively (Table 3).

There was no statistically significant difference in the frequency of movement degree between the two groups. In total, 55% of the children in the two groups showed movement that could be controlled during anesthesia, and the movement in some children reached the level that caused temporary treatment. A statistically significant difference was observed in the buccal group between the two studied phases, as the observed movement after 15 min was less than in the first stage. The percentage of children who did not move during the treatment in this group was 75%, and it was 50% in the nasal midazolam group (Table 4).

The children's behavior grades were distributed between the degree (very good: some crying and limited movement) by 45% in the buccal group and 30% in the intranasal group, and the degree (good: crying or moderate movement did not affect the treatment) by 20% and 25% in the buccal and intranasal groups, respectively. One case of failure was observed in the buccal group compared to three cases in the intranasal group, in addition to three cases, two of them in the buccal group and one in the intranasal group only received partial treatment (Table 5).

There was a significant difference between the two studied phases in the intranasal group in the sleep grades (*p* value = 0.014). There was a significant difference in the buccal sedation group between the two studied phases in the movement scale (*p* value = 0.039). The success rate of sedation was 85% in the buccal group and 80% in the intranasal group, and there was no significant difference between the two groups.

## 4. Discussion

Discussion of the null hypothesis:

• There were no statistically significant differences between the two groups in the onset time of action, the recovery time from sedation, the grades of Houpt scale “sleeping, crying, and movement,” and the success rate of sedation, and then we accept the null hypothesis.
• There were no statistically significant differences between the two studied stages when comparing the frequency of sleeping in the buccal midazolam group, the frequency of crying in the two groups, and the frequency of movement in the intranasal group, and then we accept the null hypothesis.
• There were statistically significant differences between the two studied stages when comparing the frequency of sleeping in the intranasal midazolam group, and the frequency of movement in the buccal group, and then we refused the null hypothesis.

The goal of conscious sedation in pediatric dentistry is to achieve the appropriate level of sedation with a high margin of safety [17]. Midazolam is an ideal sedative agent in pediatric dentistry because of its high safety margin, it can be applied in different ways, in addition to what it causes of amnesia and muscle relaxation [2].

This study compared the efficacy of buccal and intranasal midazolam sedation by studying the onset time of action and recovery time from sedation, behavioral response, as well as the success of sedation.

The mean onset action was 13.73 min in buccal group and 13.15 min in intranasal group, it was not expected to find a difference in the onset time between the two groups, because the two ways of sedation belong to transmucosal absorption, which has a rapid onset of action as it does not pass through the liver [18]. This result is consistent with the study of Sunbul et al. [19] where the onset time of action was similar in both groups, with an increase in the speed of action in the intranasal midazolam group.

Recovery time from sedation is defined as the interval between the end of treatment and when the patient is allowed to leave home [16]. Recovery time from sedation was 22.90 min and 23.80 min in buccal and intranasal group, respectively, with no statistically significant difference between the two groups. The variance in the recovery time from sedation with similar previous studies can be explained by the difference in the performed treatment, which was pulpotomy in this study, when it was extracted in the other studies [10, 20].

The Houpt scale was used to assess the behavioral response during procedure, this scale is useful because it classifies the evaluation of the intensity of behavioral expressions (sleeping, movement, and crying), thus increasing the opportunity to notice specific changes in the behavior of the child [21].

For the sleeping scale, there were statically significant differences in the intranasal group between the two studied phases, 65% of children were drowsy at the moment of the anesthesia, and the percent became 95% in the second phase after 15 min, the rest of children were fully awake in the two phases. One child in the buccal group fell asleep, while the rest of the children distributed between awake and drowsy without a statistically significant difference. These results confirm that midazolam is effective to reach the favorable level of sedation for dentists, when the patient is sedated, but still conscious and interacts with verbal commands.

The crying was a real challenge during the treatment procedures especially at the local anesthesia phase, 40% of children in both groups showed continuous crying during the local anesthesia, and 15% of children in intranasal group showed hysterical crying. The hysterical crying can be attributed to the paradoxical reactions that can be a result of the midazolam sedation, which include restlessness, hallucination, agitation, and inconsolable crying [22]. In total, 55% of children in the buccal and intranasal groups showed a movement which can be controlled during the local anesthesia. There were statistically significant differences in the buccal group between the two studied phases, the movement was in the second phase (after 15 min) less than the movement at local anesthesia. In total, 75% of children did not move during treatment in the buccal group, while the percent of “no movement” was 50% in the intranasal group which emphasizes the effectiveness of midazolam in reducing unwanted movements which can impede treatment.

There was one failed treatment in the buccal group sedation, while there were three failed cases in the intranasal group where the treatment could not be accomplished although local anesthesia was applied. The success rate was 85% in the buccal group, while was 80% in the intranasal group without statically significant difference; subsequently, intranasal and buccal midazolam sedation cannot be the solution for all behavioral problems.

### 4.1. Limitation of the Study

It was not possible in this study to conduct the blindness for the researcher or the patient, due to the difference in the technique of the used sedation methods, and it was applied to the evaluators who assessed the behavior of the children.

## 5. Conclusions

In the limitation of this study, each of intranasal and buccal midazolam sedation can be effective in the management of non-cooperative children during dental treatment, with a success rate of 85% in the buccal group and 80% in the intranasal group.

## Figures and Tables

**Table 1:** The Houpt sedation rating scale.

Rating scale	Definitions
Rating scale for sleep	1. Fully awake, alert 2. Drowsy, disoriented 3. Asleep, intermittent 4. Sound asleep

Rating scale for crying	1. Hysterical crying that interrupts treatment 2. Continuous, persistent crying that makes treatment difficult 3. Intermittent, mild crying that does not interfere with treatment 4. No crying

Rating scale for movement	1. Violent movement that interrupts treatment 2. Continuous movement that makes treatment difficult 3. Controllable movement that does not interfere with treatment 4. No movement

Rating scale for overall behavior	1. Aborted: no treatment 2. Poor: treatment interrupted, only partial treatment completed 3. Fair: treatment interrupted but eventually all completed 4. Good: difficult, but all treatments performed 5. Very good: some limited crying or movement, for example, during anesthesia or mouth prop 6. Excellent: no crying or movement

**Table 2:** Rating of the sleeping scale during the sedation procedure.

Drug route	Phase	Fully awake, *n* (%)	Drowsy, *n* (%)	Asleep, intermittent, *n* (%)	Sound asleep, *n* (%)	Total
Buccal	At LA	8 (40)	12 (60)	0	0	20 (100)
	After 15 m	6 (30)	13 (65)	0	1 (5)	20 (100)
Intranasal	At LA	7 (35)	13 (65)	0	0	20 (100)
	After 15 m	1 (5)	19 (95)	0	0	20 (100)

*Note:* Insignificant difference for the first phase between the two groups (*p*=0.747). Insignificant difference for the second phase between the two groups (*p*=0.115). Insignificant difference between the two studied phases in the buccal group (*p*=0.157). Significant difference between the two studied phases in the intranasal group (*p*=0.014).

Abbreviation: LA, local anesthesia.

**Table 3:** Rating of the crying scale during the sedation procedure.

Drug route	Phase	Hysterical crying, *n* (%)	Continuous crying, *n* (%)	Intermittent crying, *n* (%)	No crying, *n* (%)	Total
Buccal	At LA	0	8 (40)	7 (35)	5 (25)	20 (100)
	After 15 m	2 (10)	4 (20)	1 (5)	13 (65)	20 (100)
Intranasal	At LA	3 (15)	8 (40)	6 (30)	3 (15)	20 (100)
	After 15 m	6 (30)	1 (5)	5 (25)	8 (40)	20 (100)

*Note:* Insignificant difference for the first phase between the two groups (*p*=0.183). Insignificant difference for the second phase between the two groups (*p*=0.145). Insignificant difference between the two studied phases in the buccal group (*p*=0.102). Insignificant difference between the two studied phases in the intranasal group (*p*=0.279).

Abbreviation: LA, local anesthesia.

**Table 4:** Rating of the movement scale during the sedation procedure.

Drug route	Phase	Violent movement, *n* (%)	Continuous movement, *n* (%)	Controllable movement, *n* (%)	No movement, *n* (%)	Total
Buccal	At LA	3 (15)	2 (10)	11 (55)	4 (20)	20 (100)
	After 15 m	4 (20)	1 (5)	0	15 (75)	20 (100)
Intranasal	At LA	2 (10)	4 (20)	11 (55)	3 (15)	20 (100)
	After 15 m	5 (25)	2 (10)	3 (15)	10 (50)	20 (100)

*Note:* Insignificant difference for the first phase between the two groups (*p*=0.732). Insignificant difference for the second phase between the two groups (*p*=0.199). Significant difference between the two studied phases in the buccal group (*p*=0.039). Significant difference between the two studied phases in the intranasal group (*p*=0.439).

Abbreviation: LA, local anesthesia.

**Table 5:** Results of overall behavior scale at the end of treatment.

Drug route	Aborted, *n* (%)	Poor, *n* (%)	Fair, *n* (%)	Good, *n* (%)	Very good, *n* (%)	Excellent, *n* (%)	Total
Buccal	1 (5)	2 (10)	2 (10)	4 (20)	9 (45)	2 (10)	20 (100)
Intranasal	3 (15)	1 (5)	3 (15)	5 (25)	6 (30)	2 (10)	20 (100)

*Note:* Insignificant difference between the two groups (*p*=0.393).

## Data Availability

The data that support the findings of this study are available from the corresponding author upon reasonable request.
